# Pharmacological safety and real-world efficacy of the potassium-competitive acid blocker fexuprazan in cardiovascular patients receiving antithrombotic therapy: a prospective cohort study (FEXGARD)

**DOI:** 10.3389/fphar.2026.1738012

**Published:** 2026-06-12

**Authors:** You Mi Hwang, Sung Jung Kim, Myungjae Yoo

**Affiliations:** 1 Division of Cardiology, Department of Internal Medicine, St. Vincent’s Hospital, College of Medicine, The Catholic University of Korea, Suwon, Republic of Korea; 2 Catholic Research Institute for Medical Science, The Catholic University of Korea, Seoul, Republic of Korea

**Keywords:** antithrombic agent, clinical phamacology, CVD (cardio vascular disease), fexuprazan, GERD (gastroesophageal reflux disease), GI bleeding

## Abstract

**Background and Aims:**

Gastroesophageal reflux disease (GERD) is common in patients with CVD receiving long-term antithrombotic therapy, increasing gastrointestinal (GI) bleeding risk. Potassium-competitive acid blockers, such as fexuprazan, provide rapid and potent acid suppression; however, relevant evidence in this high-risk population is limited. This study evaluated the effects of fexuprazan on fasting serum gastrin levels, GERD symptoms, and bleeding outcomes in patients with CVD receiving antithrombotic agents.

**Methods:**

In this prospective observational study conducted at a tertiary hospital, 400 patients receiving antiplatelet or anticoagulant therapy were administered fexuprazan. Fasting venous blood samples were collected at baseline and 6 months, and total serum gastrin levels were measured. GERD symptoms were assessed using the Frequency Scale for the Symptoms of GERD (FSSG). Secondary outcomes, including GI bleeding, other-site bleeding, and mortality, were recorded over 12 months. Subgroup analyses were performed according to antithrombotic regimen and age.

**Results:**

Fexuprazan significantly increased fasting serum gastrin (152.1 ± 215.9 vs. 249.1 ± 210.3 pg/mL; p < 0.001) and improved FSSG total scores (16.3 ± 5.0 vs. 15.0 ± 6.0; p < 0.001). During the 12 months, two GI bleeding events (0.5%), 11 other-site bleeding events (2.8%), and no deaths occurred. Subgroup analyses revealed no significant differences between users of antiplatelet agents, warfarin, and non-vitamin K antagonist oral anticoagulants (NOACs). However, bleeding events occurred only in the NOAC group. Patients aged 70 years or older had a greater increase in gastrin than younger patients (+118.4 vs. +64.3 pg/mL; p = 0.025).

**Conclusion:**

Fexuprazan increased gastrin levels and improved GERD symptoms with few bleeding events in patients with CVD receiving antithrombotic therapy. This study provides clinical data on the safety and tolerability of fexuprazan in a high-risk cardiovascular population under long-term antithrombotic therapy. Elderly patients exhibited a greater increase in gastrin levels, warranting closer monitoring and further investigation.

## 1 Introduction

Gastroesophageal reflux disease (GERD) and peptic ulcer disease are among the most common acid-related disorders (Yamamichi et al., 2012; Vakil et al., 2006), and their prevalence is particularly high in patients with cardiovascular disease (CVD) receiving long-term antithrombotic therapy. Anticoagulants and antiplatelet agents substantially increase the risk of upper gastrointestinal (GI) complications, including erosion, ulcers, and bleeding (Steinberg et al., 2017; Bahit et al., 2017). In this high-risk group, optimizing acid suppression is crucial not only to control symptoms but also to prevent GI bleeding, which is associated with significant morbidity and mortality.

The GERD symptom burden can be systematically evaluated using the Frequency Scale for the Symptoms of GERD (FSSG), a validated questionnaire that correlates well with both endoscopic findings and disease severity (Kusano et al., 2004; Danjo et al., 2009). Previous studies have demonstrated that higher FSSG scores are associated not only with typical reflux symptoms but also with the presence of esophagitis and peptic ulcer disease (Danjo et al., 2009). Moreover, reflux symptoms and acid-related mucosal injuries may increase the risk of gastric ulceration and bleeding in patients receiving long-term antithrombotic therapy (Shirai et al., 2016; Baik et al., 2025). Therefore, assessing both symptom burden and endoscopic risk using validated tools, such as the FSSG, is clinically significant, as it provides an indirect marker for mucosal vulnerability and highlights the need for appropriate acid suppression therapy.

Proton pump inhibitors (PPIs) have long been the mainstay of acid suppression; however, their limitations include delayed onset, CYP2C19-dependent metabolism, and incomplete nocturnal acid control. Recently, potassium-competitive acid blockers (P-CABs), such as vonoprazan and fexuprazan, have emerged, offering rapid, potent, and sustained inhibition of gastric acid secretion (Agago et al., 2024; Ali et al., 2025; Tietto et al., 2025). Fexuprazan has demonstrated non-inferiority to esomeprazole in the treatment of erosive esophagitis (Kang et al., 2025) and may offer advantages for gastroprotection in patients at high risk of bleeding. Fexuprazan, a novel potassium-competitive acid blocker (P-CAB), differs from proton pump inhibitors (PPIs) by providing more rapid and sustained acid suppression, minimal influence from CYP2C19 polymorphisms, and more consistent intragastric pH control.

However, significant gaps remain in the evidence. Although P-CABs have demonstrated efficacy in erosive esophagitis and in preventing aspirin-associated ulcers (Yang, 2020; Ahn, 2020; An et al., 2024), data on their effects in patients with CVD are lacking. In particular, the dynamics of serum gastrin under P-CAB treatment, its relationship with reflux symptom outcomes, and its impact on bleeding risk remain unclear (Schubert, 2008; Shiotani et al., 2018; Lee et al., 2019; Jung et al., 2025).

Cardiovascular patients on long-term antithrombotic therapy are frequently underrepresented in trials assessing acid-suppressive agents. This study addresses an unmet clinical need by focusing on patients who often experience reflux-related intolerance during essential antithrombotic treatment, potentially leading to therapy discontinuation. The present study aimed to evaluate whether fexuprazan could improve reflux-related symptoms while maintaining safety in this clinically vulnerable population.

## 2 Materials and methods

### 2.1 Study design and setting

This prospective, single-center, observational study (FEXGARD) was conducted at St. Vincent’s Hospital, College of Medicine, The Catholic University of Korea. This study evaluated the association between serum gastrin levels and GERD symptoms before and after fexuprazan administration in patients with CVD receiving antithrombotic therapy. The study protocol was approved by the Institutional Review Board (IRB), and written informed consent was obtained from all participants in accordance with the principles outlined in the Declaration of Helsinki (IRB No. VC24OISI0024).

### 2.2 Study population

Adult patients (≥20 years) with CVD who were prescribed at least one anticoagulant or antiplatelet agent and had symptomatic or endoscopic evidence of GERD were eligible for enrollment. The inclusion criteria required that participants were newly prescribed fexuprazan within the preceding 2 months, to ensure inclusion of patients in the early phase of treatment and to minimize variability related to long-term exposure and were able to provide informed consent. The exclusion criteria were as follows: (1) life expectancy <6 months, (2) cognitive impairment precluding consent, (3) prior history of fexuprazan use exceeding 3 months, and (4) withdrawal of consent during follow-up. A target sample size of 400 patients was planned based on feasibility considerations and expected recruitment during the study period, rather than a formal power calculation, given the exploratory nature of the study, with a potential 10% dropout rate accounted for. All patients received fexuprazan 40 mg once daily for 12 months.

### 2.3 Blood sampling and gastrin measurement

Fasting venous blood samples were collected in the morning after an overnight fast of 8–12 h (baseline) and before the daily dose of fexuprazan to minimize postprandial variation. Serum was separated within 2 h of collection and analyzed in the central clinical laboratory of St. Vincent’s Hospital. Total serum gastrin levels were quantified using the IMMULITE Gastrin chemiluminescent immunoassay on an IMMULITE 2000/2000 XPi analyzer (Siemens Healthineers, Erlangen, Germany) under fasting conditions to measure the overall circulating gastrin isoforms. For consistency, all paired samples from the same participant (baseline and 6-month follow-up) were run in the same analytical batch to minimize inter-assay variation and prevent hemolysis.

### 2.4 GERD symptom and baseline gastrointestinal medication assessment

GERD symptoms were assessed using the FSSG questionnaire at baseline and after the initiation of fexuprazan. The FSSG consists of 12 items assessing reflux-related (7 items) and dysmotility-related (5 items) symptoms, each rated on a 5-point Likert scale (0–4), with higher total scores indicating more severe symptom burden. Total scores and subscores (reflux- and dysmotility-related) were recorded and analyzed.

Baseline gastrointestinal medication assessments were also conducted at the time of study enrollment; these included PPIs, H_2_ receptor antagonists, and mucosal protectants (rebamipide and sucralfate).

### 2.5 Study outcomes

The primary endpoints were as follows: (1) change in fasting total serum gastrin levels from baseline to 6 months, and (2) change in GERD symptom burden as assessed by the FSSG scores.

The secondary endpoints were the incidence of anemia, GI bleeding requiring transfusion, defined as clinically overt bleeding associated with a decrease in hemoglobin ≥2 g/dL, need for transfusion, or requiring endoscopic or surgical intervention, and all-cause mortality (classified as cardiovascular or non-cardiovascular) during the 12-month follow-up period.

### 2.6 Statistical analysis

Continuous variables are expressed as mean ± standard deviation and were compared using Student’s t-test or Wilcoxon rank-sum test, as appropriate. Categorical variables are expressed as numbers (percentages) and were compared using the chi-square test or Fisher’s exact test. Paired analyses were performed for within-patient changes in gastrin levels and FSSG scores. Kaplan–Meier survival curves were constructed to assess bleeding-free survival, and comparisons were performed using the log-rank test. Subgroup analyses were conducted using antithrombotic regimens and patient age (<70 vs. ≥70 years). All statistical analyses were performed using R version 4.5.1 (R Foundation for Statistical Computing, Vienna, Austria), and a two-sided P < 0.05 was considered statistically significant. All statistical analyses were performed independently by the investigators without sponsor involvement. Given the limited number of bleeding events, results were presented descriptively rather than inferentially.

### 2.7 Ethics

The study protocol was reviewed and approved by the Institutional Review Board of St. Vincent’s Hospital, The Catholic University of Korea (approval no. VC24OISI0024). Written informed consent was obtained from all participants before enrollment, and the study was conducted in accordance with the principles outlined in the Declaration of Helsinki.

## 3 Results

### 3.1 Baseline characteristics

A total of 400 patients with CVD who received antithrombotic therapy were enrolled in this study. The mean age was 72.2 ± 10.5 years, and 58.2% were male. The mean body mass index was 24.4 ± 3.9 kg/m^2^. Regarding antithrombotic therapy, 7.5% of the patients received antiplatelet agents, 2.5% received warfarin, and 91.8% received non-vitamin K antagonist oral anticoagulants (NOACs) with percentages exceeding 100% due to overlapping antithrombotic therapies (e.g., combination regimens). The most frequent comorbidity was atrial fibrillation (95.0%), followed by coronary artery disease (15.8%), chronic kidney disease (18.5%), and heart failure (32.0%). Table 1 summarizes the baseline demographic and clinical characteristics of patients. Of 400 initially enrolled participants, 367 completed the 12-month follow-up (91.8% retention). Thirty-three participants discontinued due to non-study-related reasons (loss to follow-up or medication change).

**TABLE 1:** Baseline characteristics of the study population (n = 400).

Variable	Value
Age (years)	72.2 ± 10.5
Male sex	233 (58.2%)
Female sex	167 (41.8%)
BMI (kg/m^2^)	24.4 ± 3.9
Antiplatelet use	30 (7.5%)
Warfarin use	10 (2.5%)
NOAC use	367 (91.8%)
Atrial fibrillation	380 (95.0%)
Coronary artery disease	63 (15.8%)
Peripheral artery disease	2 (0.5%)
COPD	15 (3.8%)
Chronic kidney disease	74 (18.5%)
Heart failure	128 (32.0%)

### 3.2 Changes in fasting serum gastrin levels

**FIGURE 1:**
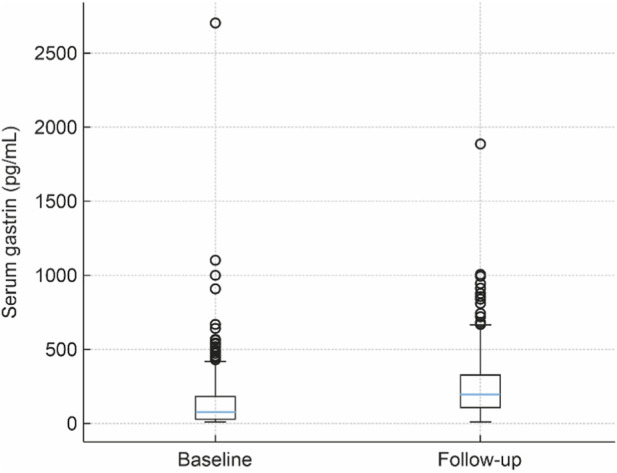
Change in fasting serum gastrin levels before and after fexuprazan treatment. Box plots represent median, interquartile range, and outliers. P < 0.001 by paired t-test.

Among 367 paired measurements, fasting total serum gastrin levels significantly increased after 6 months of fexuprazan therapy (152.1 ± 215.9 pg/mL vs. 249.1 ± 210.3 pg/mL, Δ +97.0 pg/mL; p < 0.001). The results are presented in Table 2 and illustrated in Figure 1.

**TABLE 2:** Overall changes in fasting serum gastrin and FSSG total score from baseline to follow-up.

Outcome variable	Baseline mean ± SD	Follow-up mean ± SD	Δ change	P-value
Serum gastrin (pg/mL)	152.1 ± 215.9	249.1 ± 210.3	97.0	<0.001
FSSG total score	16.3 ± 5.0	15.0 ± 6.0	−1.2	<0.001

### 3.3 Changes in GERD symptoms (FSSG scores)

In 367 patients with paired assessments, GERD symptoms significantly improved after initiation of fexuprazan, with mean FSSG total scores decreasing from 16.3 ± 5.0 to 15.0 ± 6.0 (Δ −1.2; p < 0.001). Both reflux- and dysmotility-related subscores demonstrated similar trends. Table 2 presents the results on changes in the FSSG scores.

### 3.4 Secondary outcomes

During the 12-month follow-up, no drug-related adverse events such as dyspepsia, diarrhea, or hepatic enzyme elevation were reported. Two patients (0.5%) experienced GI bleeding events, 11 patients (2.8%) developed bleeding at other sites, and no deaths were reported. The data are summarized in Table 3. No signal suggesting an increased bleeding tendency was observed during fexuprazan therapy. Among patients who experienced bleeding events, most had multiple comorbidities, including advanced age, atrial fibrillation, and concomitant anticoagulant therapy, suggesting that bleeding risk was primarily driven by underlying clinical characteristics rather than fexuprazan itself. Even among patients receiving dual antiplatelet or anticoagulant regimens, post-treatment laboratory indices and hemoglobin remained stable. Liver function tests demonstrated small but statistically significant increases in mean AST (29.3 ± 14.1 U/L to 34.1 ± 24.4 U/L, p = 0.001) and ALT (24.4 ± 17.2 U/L to 28.7 ± 22.1 U/L, p = 0.003) after 6 months of fexuprazan therapy. However, all values remained within the normal laboratory range, and no patient experienced an elevation exceeding three times the upper limit of normal. These mild changes were not accompanied by any clinical symptoms or treatment discontinuation, suggesting limited clinical significance (≤1.2× ULN in all cases).

**TABLE 3:** Incidence of gastrointestinal (GI) and non-GI bleeding events and deaths during 12 months of follow-up.

Outcome	n (%)
GI bleeding events	2 (0.5%)
Other site bleeding events	11 (2.8%)
Deaths	0 (0.0%)

### 3.5 Kaplan-Meier analysis of bleeding-free survival

Kaplan–Meier curves showed that GI bleeding-free survival remained >99% during the 12-month follow-up period, with single events observed at 6 and 12 months. In contrast, the bleeding-free survival rates at other sites showed more pronounced declines, with events observed at 3, 6, and 12 months.

**FIGURE 2:**
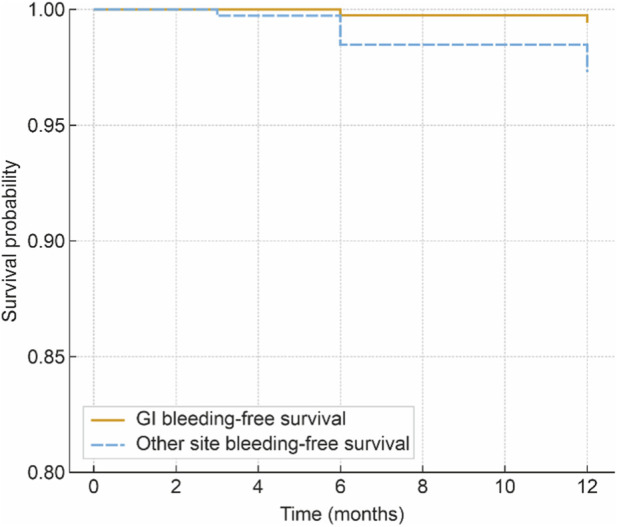
Kaplan–Meier curves for gastrointestinal (GI) versus non-GI bleeding events during 12 months of follow-up. Log-rank test, P = NS. GI, gastrointestinal.

The curves are shown in Figure 2, with the y-axis restricted to 0.80–1.00 to emphasize event-related declines.

### 3.6 Subgroup analyses by antithrombotic therapy

Subgroup analyses were used to compare outcomes among patients receiving antiplatelet therapy (n = 22), warfarin (n = 10), and NOACs (n = 367). The mean change in fasting serum gastrin was numerically highest in the warfarin group (+194.0 ± 162.0 pg/mL), followed by the antiplatelet (+137.3 ± 210.3 pg/mL) and NOAC (+92.6 ± 247.3 pg/mL) groups. However, these differences were not significant (ANOVA, p = 0.365). For GERD symptoms, the mean change in FSSG total scores was −3.1 ± 5.9, −2.5 ± 5.1, and −1.1 ± 5.6 in the antiplatelet, warfarin, and NOAC groups, respectively, with no significant difference among the groups (ANOVA, p = 0.207). Bleeding events were observed only in the NOAC group (2 GI bleeding events and 11 other-site bleeding events), whereas no such events occurred in the warfarin or antiplatelet groups. However, the small sample sizes of the warfarin and antiplatelet groups limited statistical comparisons. These results are summarized in Supplementary Table S1 and shown in Supplementary Figure S1.

### 3.7 Subgroup analyses by age

When stratified by age, patients aged ≥70 years demonstrated a significantly greater increase in fasting total serum gastrin than those aged <70 years (+118.4 ± 271.9 vs. +64.3 ± 189.4 pg/mL, p = 0.025). In contrast, improvements in FSSG total scores did not differ significantly between the two groups (−1.5 ± 5.7 vs. −0.8 ± 5.5, p = 0.182). Bleeding events were infrequent and occurred in both age groups (≥70 years: 2 GI bleeding, seven other-site bleeding events; <70 years: four other-site bleeding events).

These results are summarized in Supplementary Table S2 and illustrated in Supplementary Figure S2.

### 3.8 Subgroup analyses by previous GI medication

In the subgroup analysis stratified by baseline GI medication use, patients receiving GI medications (n = 258) had significantly higher baseline gastrin levels than those not receiving GI medications (176.3 ± 176.4 vs. 107.5 ± 259.1 pg/mL, *p* = 0.002). The baseline FSSG total scores were comparable between groups. Following fexuprazan treatment, gastrin levels significantly increased in both subgroups (no GI medications: 107.5–245.3 pg/mL, *p* < 0.001; GI medications: 176.3–251.3 pg/mL, *p* < 0.001), while FSSG scores significantly decreased (no GI medications: 16.1 to 14.2, *p* < 0.001; GI medications: 16.4 to 15.5, *p* < 0.01). These results indicate that symptom improvement was consistent regardless of prior GI medication use, although baseline gastrin levels were higher in patients who had been previously treated. These findings, summarized in Supplementary Table S3 and Supplementary Figure S3, align with the overall results, indicating that the therapeutic effects of fexuprazan on gastrin elevation and symptom improvement were consistent regardless of prior GI medication use.

## 4 Discussion

### 4.1 Principal findings

This prospective observational study demonstrated that fexuprazan therapy significantly increased total fasting serum gastrin levels and improved GERD symptoms in patients with CVD receiving antithrombotic agents. Bleeding events were infrequent, and no deaths occurred during the 12 months of follow-up. Subgroup analyses revealed no significant differences in gastrin levels or symptom outcomes between the antiplatelet, warfarin, and NOAC groups. However, bleeding events were observed exclusively in the NOAC cohort, consistent with prior trials of anticoagulants and antiplatelet agents (Bahit et al., 2017; Giugliano et al., 2013; Caldeira et al., 2015; Granger et al., 2011; Dans et al., 2013; Lip et al., 2025; Patel et al., 2011). Multivariable adjustment was not performed due to the low number of bleeding events. Importantly, age-based analyses revealed that patients aged 70 years or older experienced a significantly greater rise in fasting serum gastrin compared to younger patients. The improvements in GERD symptoms were similar across all age groups. From a clinical pharmacology perspective, these findings highlight the favorable hepatic and gastrointestinal safety profile of fexuprazan under real-world, polypharmacy conditions.

### 4.2 Comparison with previous studies

Our findings align with those of previous reports on PPI- and P-CAB-induced hypergastrinemia and confirm that fexuprazan exerts a similar effect in high-risk patients with CVD (Shiotani et al., 2018; Jung et al., 2025). Prior GERD trials demonstrated symptomatic efficacy, and our study extends this evidence to an elderly population with CVD receiving antithrombotic therapy (Agago et al., 2024; An et al., 2024; Miyamoto et al., 2010; Komatsu-Tanaka et al., 2012).

The subgroup analyses provided novel insights. While prior studies have rarely compared outcomes across antithrombotic regimens, we observed no significant differences among users of antiplatelet agents, warfarin, and NOACs. However, bleeding events were more prevalent in the NOAC group; the predominance of NOAC use and the small sample sizes in other groups limit direct comparison and may influence the detection of adverse events. Although gastrin elevation was more pronounced in elderly patients (≥70 years), no case of gastrointestinal bleeding was observed during the 6-month follow-up period. This suggests that fexuprazan may be safe even in elderly high-risk patients, although careful follow-up and long-term surveillance remain essential in this population.

Beyond the antithrombotic regimen and age, baseline GI medication use has emerged as another determinant of gastrin profile. Patients previously exposed to acid suppressants exhibited higher baseline gastrin levels, which is consistent with compensatory hypergastrinemia. However, fexuprazan increased gastrin levels and improved GERD symptoms in both groups, underscoring its robust efficacy across diverse patient populations. Clinically, this suggests that switching from conventional acid suppressants to fexuprazan, or initiating fexuprazan *de novo*, may provide consistent symptomatic benefits to diverse patient populations. However, the finding of elevated baseline gastrin levels in the GI medication subgroup underscores the importance of long-term monitoring for potential hypergastrinemia-related sequelae in this group.

### 4.3 Clinical implications

These findings support the integration of fexuprazan into routine clinical practice for patients receiving long-term antithrombotic therapy who are at elevated risk of GI complications. However, clinicians should be aware of the greater increase in gastrin levels in older patients, which may warrant closer monitoring during long-term therapy. The bleeding events were observed primarily among NOAC users; however, given the unequal group sizes, this should not be interpreted as evidence of no risk in other antithrombotic groups. Given that patients requiring chronic antithrombotic therapy are intrinsically predisposed to gastrointestinal bleeding, the absence of excess bleeding with fexuprazan provides particularly reassuring evidence of safety in this vulnerable population.

The clinical significance of hypergastrinemia remains uncertain. While gastrin elevation did not correlate with symptom worsening or bleeding in our study, chronic hypergastrinemia has been linked to ECL-cell hyperplasia in experimental models. Long-term follow-up studies are warranted to clarify its physiological implications in elderly patients receiving antithrombotic therapy. In the present study, elevations in fasting serum gastrin levels were accompanied by symptomatic improvement, suggesting that short-term hypergastrinemia may reflect effective acid suppression and thus be protective against GERD symptoms. However, chronic or sustained hypergastrinemia has been associated with gastric mucosal hypertrophy, polyps, and, rarely, neuroendocrine tumors (Shiotani et al., 2018; Lee et al., 2019; Yamaguchi et al., 2017; Ness-Jensen et al., 2021). Therefore, although short-term increases in gastrin levels appear to be beneficial as a pharmacodynamic marker of drug efficacy, long-term monitoring may be warranted, particularly in elderly patients who exhibited more pronounced gastrin elevation in our study.

While the pharmacological effects of PCABs, including fexuprazan, on gastrin elevation and reflux control are established, our study uniquely addresses a high-risk cardiovascular cohort receiving antithrombotic therapy. This group is underrepresented in prior clinical studies, despite their heightened vulnerability to gastrointestinal complications. Notably, the consistent improvement in GERD symptoms across subgroups, along with the absence of an increased bleeding risk, provides clinically meaningful reassurance. Fexuprazan may allow safer continuation of antithrombotic therapy by alleviating reflux symptoms without compromising gastrointestinal safety. The modest rise in serum gastrin observed in our study is consistent with the known physiological adaptation to acid suppression. It remains well below the pathological range associated with enterochromaffin-like cell hyperplasia. The observed increase in serum gastrin likely reflects physiological adaptation to acid suppression (Schubert, 2008). As illustrated in Supplementary Figure S4, fexuprazan-induced acid inhibition enhances gastrin secretion via G-cell stimulation, which transiently promotes ECL-cell hypertrophy before a negative feedback mechanism stabilizes gastrin levels within the normal range. This schematic representation highlights the adaptive feedback loop underlying gastrin homeostasis during fexuprazan therapy. Similar findings were reported in prior PPI studies (Shiotani et al., 2018), but our study expands this evidence to patients receiving concurrent antithrombotic treatment. Such adaptive feedback may partly explain the favorable balance between efficacy and safety observed in our cohort. No signal of increased bleeding risk was observed, even in those using dual antiplatelet therapy. These findings have broad relevance in regions such as Korea, where P-CABs are increasingly replacing PPIs in clinical practice. Although the reduction in total FSSG score was statistically significant, the absolute change (1–2 points) may not represent a clinically meaningful improvement. Nevertheless, the observed trend suggests symptom stabilization and tolerability in a high-risk cardiovascular population, where even minor relief can impact quality of life. It may translate into quality of life and adherence to essential antithrombotic therapy. Improved symptom control may also enhance adherence to long-term antithrombotic therapy, which is often compromised by gastrointestinal discomfort.

Although mild elevations in AST and ALT were observed, the magnitude of change was minimal and clinically insignificant, with all values remaining within normal limits. The absence of symptomatic hepatotoxicity or treatment withdrawal indicates that these enzyme fluctuations likely reflect physiological variability rather than drug-induced liver injury. Unlike several proton pump inhibitors that undergo extensive hepatic CYP metabolism, fexuprazan’s minimal CYP2C19 dependence may contribute to its favorable hepatic safety profile. Given that many cardiovascular patients are concomitantly exposed to hepatically metabolized agents such as statins or amiodarone, this finding further supports the hepatic tolerability of fexuprazan in multimorbid settings. Fexuprazan is not significantly influenced by CYP2C19 metabolism and has been reported in previous pharmacological studies of P-CABs, indicating minimal known pharmacokinetic interactions with antiplatelet or anticoagulant agents and a low likelihood of drug–drug interactions affecting clinical outcomes.

### 4.4 Study limitations

This study has some limitations. First, its single-center observational design limits generalizability and prevents causal inferences. This study did not include a conventional PPI control group, as it was designed as a prospective observational study reflecting routine clinical practice. This limits direct comparison and causal interpretation, and the findings should therefore be considered exploratory. The pre–post within-patient design was used in the context of real-world prescribing, where a standardized alternative treatment pathway is often not available in this high-risk population. Despite this limitation, the study still provides clinically relevant information on treatment response and safety. Second, although gastrin was measured, only total serum gastrin was available, lacking isoform-specific assays like gastrin-17, which could have clarified the mechanistic pathways. Third, the subgroup analyses were limited by the small numbers of patients in the warfarin and antiplatelet groups, which reduced statistical power. Fourth, the limited number of bleeding events restricts the statistical power of subgroup analyses, and the findings should be interpreted with caution. In our cohort, systematic endoscopic evaluation was not performed, and therefore, the direct impact of fexuprazan on pre-existing mucosal erosions or ulcers could not be confirmed. Nevertheless, no ulcer-related adverse events or bleeding episodes were observed clinically during follow-up. This suggests that, despite the observed increase in gastrin, fexuprazan did not aggravate clinically significant mucosal injury within the study period. Future studies incorporating endoscopic outcomes are warranted to further clarify whether fexuprazan may contribute to mucosal healing or protection in high-risk patients.

Fifth, another consideration is the potential effect of long-term potent acid suppression on gastrointestinal function and gut microbiota. While our study did not assess digestive functional parameters or alterations in the microbiome, prior literature indicates that profound acid inhibition can influence gastric motility and microbial composition. Although no clinically significant dyspeptic symptoms or infections were reported in our cohort, future prospective studies should incorporate assessments of gastrointestinal physiology and microbiome balance to fully establish the safety profile of fexuprazan, especially in elderly patients on chronic antithrombotic therapy. Finally, follow-up was limited to 12 months; thus, longer-term safety and efficacy data are required. Age subgroup analyses should also be interpreted cautiously, as residual confounding factors (frailty, comorbidity burden, and polypharmacy) may have influenced gastrin responses in older patients.

Although this single-center real-world design provides valuable practical insight, generalizability to other populations and healthcare systems may be limited. While the present data are encouraging, larger multicenter randomized controlled trials are necessary before routine clinical use of fexuprazan in long-term antithrombotic users can be recommended. As this was a single-arm observational study without a control group, the findings demonstrate associations rather than causality. The results should therefore be interpreted as exploratory and hypothesis-generating rather than confirmatory.

### 4.5 Future directions

Future trials should evaluate fexuprazan across various antithrombotic regimens and stratify them by age to confirm whether elderly patients are more prone to hypergastrinemia. Future trials should also incorporate endoscopic assessments of mucosal healing and detailed analyses of gastrointestinal microbiota to clarify the long-term functional and ecological safety of fexuprazan in elderly patients with cardiovascular disease.

## 5 Conclusion

Fexuprazan provides effective reflux symptom relief and clinical safety in antithrombotic users, supporting its role as a rational acid-suppressive option in modern cardiovascular care.

## Data Availability

The raw data supporting the conclusions of this article will be made available by the authors, without undue reservation.
