# Supplementary material for: Pharmacological safety and real-world efficacy of the potassium-competitive acid blocker fexuprazan in cardiovascular patients receiving antithrombotic therapy: a prospective cohort study (FEXGARD)
Source: Front Pharmacol. 2026 Jun 12;17:1738012. doi: 10.3389/fphar.2026.1738012 (PMC13299093; doi:10.3389/fphar.2026.1738012)
Supplement: Supplementary file 1 [file Supplementaryfile1.docx]

**Supplementary Table 1.**

| **group** | **n** | **Δ Gastrin (mean ± SD)** | **Δ FSSG (mean ± SD)** | **GI_bleed** | **other_bleed** | **deaths** |
| --- | --- | --- | --- | --- | --- | --- |
| Antiplatelet | 22 | 137.3 ± 210.3 | -3.1 ± 5.9 | 0 | 0 | 0 |
| NOAC | 367 | 92.6 ± 247.3 | -1.1 ± 5.6 | 2 | 11 | 0 |
| Warfarin | 10 | 194.0 ± 162.0 | -2.5 ± 5.1 | 0 | 0 | 0 |

Subgroup analysis according to antithrombotic regimen (antiplatelet, NOAC, warfarin): changes (Δ) in fasting serum gastrin and FSSG total score from baseline to follow-up, and bleeding events and deaths. Values are presented as mean ± SD or number (%). Δ denotes post minus baseline.

**Supplementary Table 2.**

| **Age group** | **n** | **Δ Gastrin (mean ± SD, pg/mL)** | **Δ FSSG (mean ± SD)** | **GI bleeding (%)** | **Other bleeding (%)** | **Deaths n (%)** |
| --- | --- | --- | --- | --- | --- | --- |
| <70 yrs | 155 | +64.3 ± 189.4 | –0.8 ± 5.5 | 0 (0.0%) | 4 (2.6%) | 0 (0.0%) |
| ≥70 yrs | 245 | +118.4 ± 271.9 | –1.5 ± 5.7 | 2 (0.8%) | 7 (2.9%) | 0 (0.0%) |

Subgroup analysis by age (<70 years vs. ≥70 years): changes (Δ) in fasting serum gastrin and FSSG total score from baseline to follow-up, and bleeding events and deaths. Values are presented as mean ± SD or number (%). Δ denotes post minus baseline.

**Supplementary Table 3.**

| **GI med** | **N** | **Baseline Gastrin (Mean ± SD)** | **Post Gastrin (Mean ± SD)** | **p (Gastrin)** | **Baseline FSSG (Mean ± SD)** | **Post FSSG (Mean ± SD)** | **p (FSSG)** |
| --- | --- | --- | --- | --- | --- | --- | --- |
| 0 (No) | 142 | 107.46 ± 259.12 | 245.25 ± 234.68 | <0.001 | 16.11 ± 5.30 | 14.20 ± 5.58 | <0.001 |
| 1 (Yes) | 258 | 176.29 ± 176.35 | 251.25 ± 196.27 | <0.001 | 16.36 ± 4.90 | 15.51 ± 6.13 | <0.01 |

Subgroup analysis according to baseline GI medication use (GI med = 0, no; GI med = 1, yes): baseline and post-treatment fasting serum gastrin and FSSG total score, with within-group P values from paired t-tests. Values are presented as mean ± SD.

Abbreviations: FSSG, Frequency Scale for the Symptoms of GERD; NOAC, non-vitamin K antagonist oral anticoagulant; GI, gastrointestinal.

**Supplementary Figure 1.**


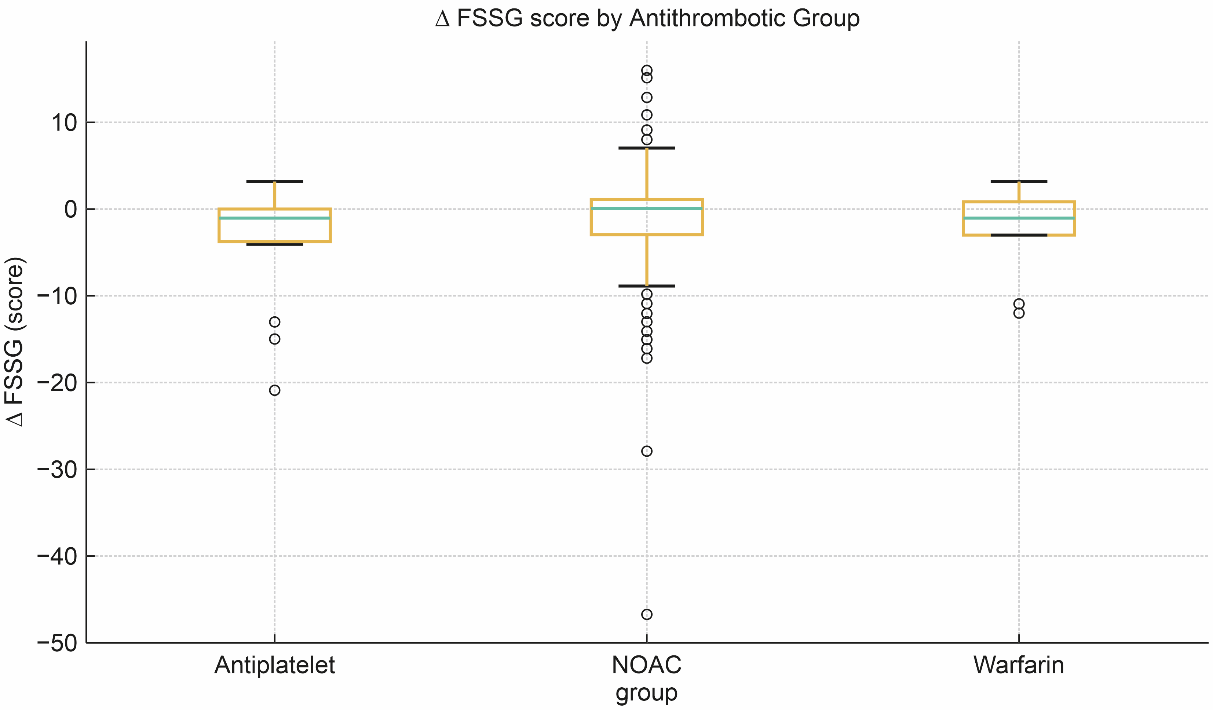


Changes in FSSG total score (ΔFSSG) according to antithrombotic regimen (antiplatelet, NOAC, warfarin). Box plots represent median, interquartile range, and outliers.

**Supplementary Figure 2.**


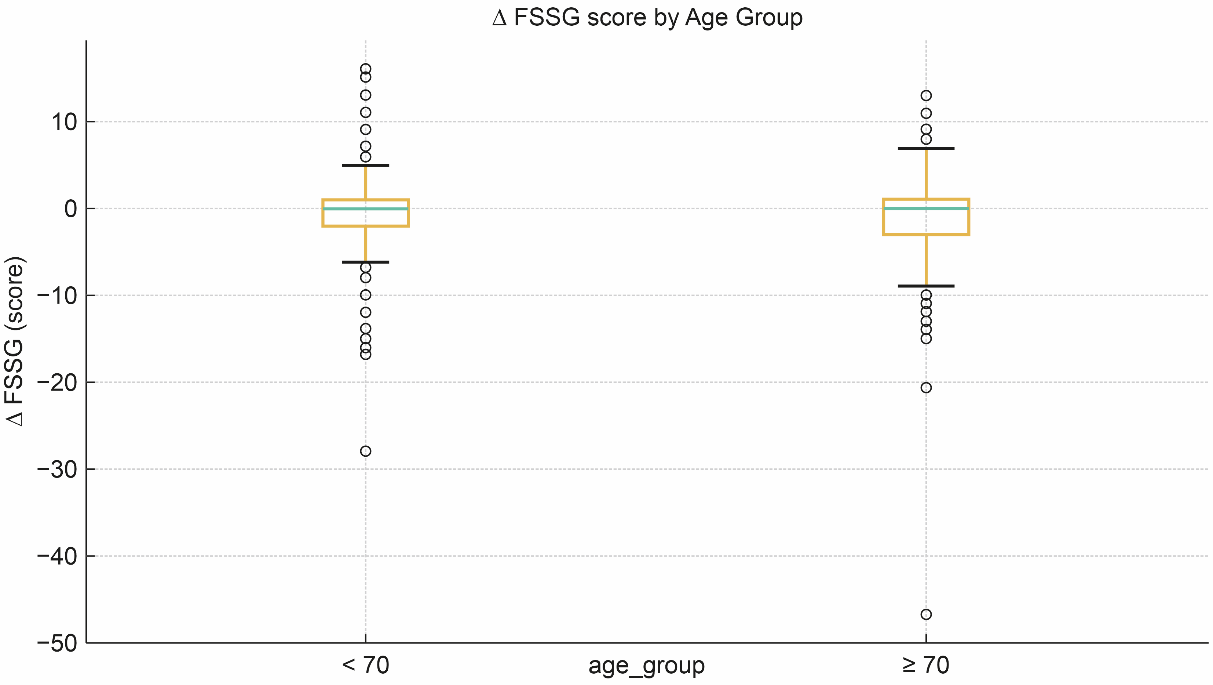


Subgroup analysis of changes in FSSG total score (ΔFSSG) stratified by age (<70 years vs. ≥70 years).

Box plots represent median, interquartile range, and outliers.

**Supplementary Figure 3.**


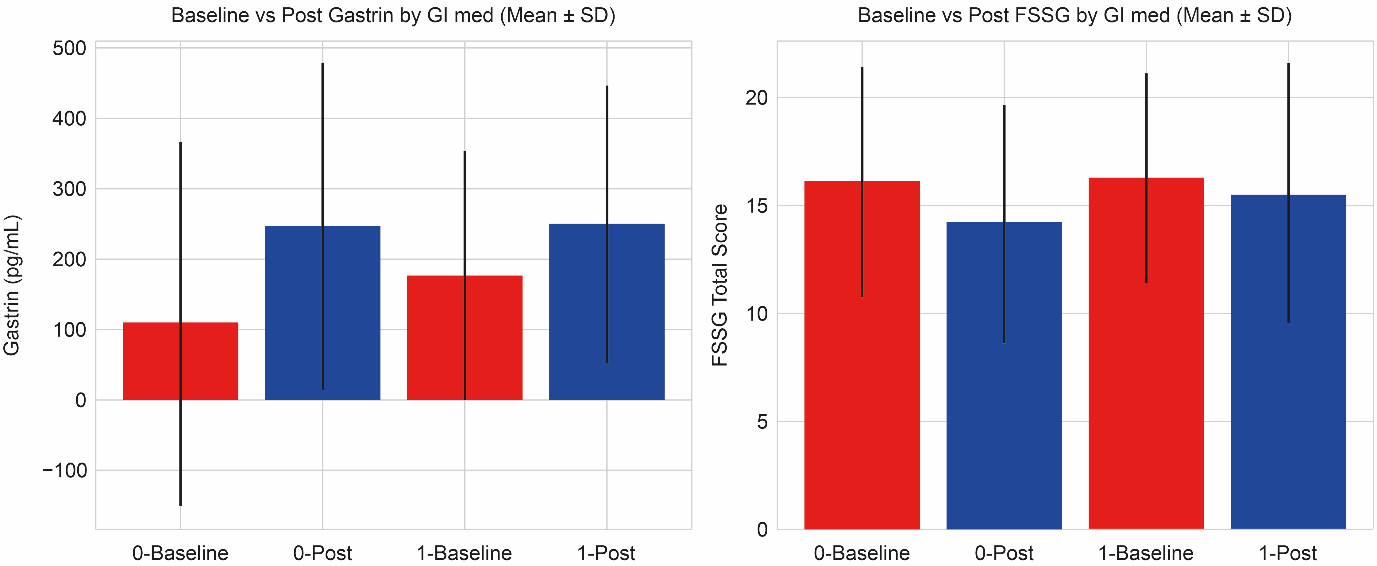
Subgroup analysis of fasting serum gastrin (left) and FSSG total score (right) according to baseline GI medication use (GI med = 0, no; GI med = 1, yes). Bars represent mean ± SD.

FSSG, Frequency Scale for the Symptoms of GERD; GERD, gastroesophageal reflux disease; GI, gastrointestinal

**Supplementary Figure 4.**

**
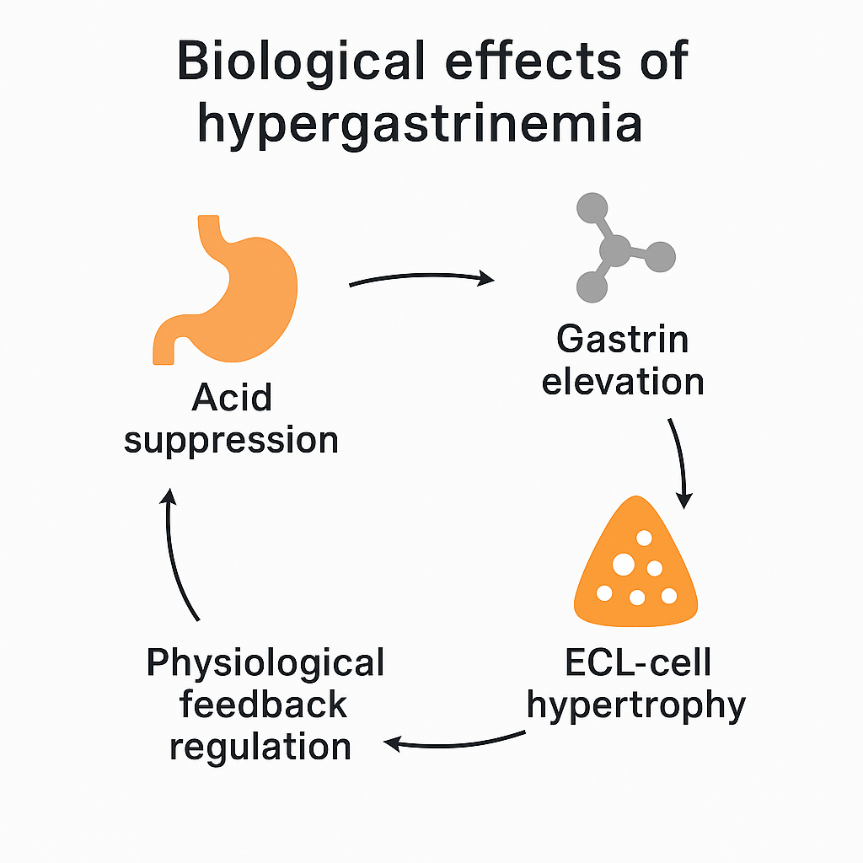
**

Schematic illustration of the biological response to acid suppression. Reduced gastric acid secretion stimulates G-cell activity, resulting in increased gastrin release, enterochromaffin-like (ECL) cell hypertrophy, and feedback regulation that maintains gastrin within the physiological range.
